# Brazilian expert consensus on the diagnosis, classification, screening for complications and treatment of familial partial lipodystrophy

**DOI:** 10.1186/s13098-025-01733-5

**Published:** 2025-06-02

**Authors:** Cynthia Melissa Valerio, Luiz F. Viola, Natália Rossin Guidorizzi, Josivan Gomes Lima, Amélio F. Godoy-Matos, Alexandre Hohl, Fabio R. Trujilho, Joana R. Dantas, Julliane Tamara Araújo de Melo Campos, Lenita Zajdenverg, Raquel Beatriz Gonçalves Muniz, Rodrigo Oliveira Moreira, Virgínia Oliveira Fernandes, Maria Cristina Foss-Freitas, Renan Montenegro

**Affiliations:** 1 Brazilian Group for the Study of Inherited and Acquired Lipodystrophies (BRAZLIPO), Fortaleza, Brazil; 2 Department of Metabolism, Institute of Diabetes and Endocrinology of Rio de Janeiro (IEDE), Rio de Janeiro, Brazil; 3 Diabetes and Endocrinology Center (CEDERO), Rondonópolis, Brazil; 4 Division of Endocrinology and Metabology, Department of Internal Medicine, Clinical Hospital of Ribeirão Preto Medicine School, University of São Paulo-Ribeirão Preto, Ribeirão Preto, Brazil; 5 Department of Internal Medicine, Hospital Universitário Onofre Lopes, Universidade Federal do Rio Grande do Norte, Rio Grande do Norte, Brazil; 6 Division of Endocrinology and Metabolism, Department of Internal Medicine, Federal University of Santa Catarina (UFSC), Florianopolis, SC Brazil; 7 Research Unit, Hospital da Obesidade, Salvador, BA Brazil; 8 Internal Medicine Department, Nutrology and Diabetes Unit, Universidade Federal do Rio de Janeiro, Rio de Janeiro, Brazil; 9 Laboratório de Genética Molecular e Metabolismo (LabGeM), Centro de Biociências, Instituto de Medicina Tropical d Rio Grande do Norte (IMT-RN), Universidade Federal do Rio Grande do Norte, Natal, RN Brazil; 10 Faculdade de Medicina de Valença, Centro Universitário de Valença, Valença, RJ Brazil; 11 Faculdade de Medicina da Universidade, Centro Universitário Presidente Antônio Carlos (FAME/UNIPAC), Juiz de Fora, MG Brazil; 12 Clinical Research Unit, Walter Cantídio University Hospital, Federal University of Ceará/EBSERH, Fortaleza, Brazil; 13 Division of Metabolism, Endocrinology and Diabetes (MEND), Department of Internal Medicine, Michigan Medicine, University of Michigan-Ann Arbor, Ann Arbor, MI USA

**Keywords:** Hypertriglyceridemia, Insulin resistance, Lipodystrophy, Diagnosis, Genetics, Therapy, Pancreatitis

## Abstract

**Background:**

Partial lipodystrophies are a rare and heterogeneous group of diseases characterized by variable loss of adipose tissue. Around the world, the high heterogeneity in phenotypic expression, limited awareness, and absence of standardized diagnostic criteria for familial partial lipodystrophies (FPLD) may contribute to the underdiagnosis of genetic forms. The estimated high prevalence of FPLD in Brazil, combined with resource limitations in the healthcare system and a lack of specialized medical centers, presents significant challenges in the diagnosis and treatment of lipodystrophy-related conditions. This expert consensus aimed to establish clinical criteria for FPLD suspicion and diagnosis, propose a flowchart for clinical and complementary evaluation, and provide a framework for managing FPLD-related disorders and complications.

**Methods:**

A consensus was reached following discussions with 15 experts from Brazilian lipodystrophy referral centers specializing in the diagnosis and management of partial lipodystrophies. Using a combination of face-to-face meetings and online and offline activities, the panel addressed five key aspects of FPLD management: clinical suspicion and diagnosis of the condition, classification of the most common subtypes, multisystem manifestations, screening for complications, and therapeutic approaches.

**Results:**

Two clinical criteria were proposed for the suspicion of FPLD: one mandatory criterion, characterized by lipoatrophy in the lower limbs, and at least one of the following conditions associated with FPLD: hypertriglyceridemia and/or low high-density lipoprotein cholesterol, diabetes mellitus, impaired fasting glucose or glucose intolerance, metabolic-associated steatosis liver disease, early coronary atherosclerotic disease, acanthosis nigricans, and polycystic ovary syndrome. To confirm the diagnosis, different combinations of criteria were suggested: presence of the mandatory criterion along with either two major criteria, one major and two minor criteria, or a positive genetic test with a mandatory criterion.

**Conclusions:**

This expert consensus provides a feasible guide based on signs of lipoatrophy and metabolic abnormalities observed in Brazilian centers of lipodystrophy to enhance clinical suspicion and enable early diagnosis of FPLD. Through adequate screening for FPLD-related complications, a therapeutic approach has been proposed that includes lifestyle modifications, early interventions for comorbidities, and targeted pharmacological treatment.

## Background

Partial lipodystrophies (PL) comprise a rare and heterogeneous group of diseases characterised by variable adipose tissue loss, with either genetic or acquired origins [1, 2]. The reduction of subcutaneous adipose tissue results in ectopic fat deposition in organs such as the liver, muscles, pancreas, and epicardial tissue. This phenomenon contributes to insulin resistance and various metabolic complications, including diabetes mellitus, hypertriglyceridemia, and metabolic-associated steatosis liver disease (MASLD). Additionally, it is associated with an increased risk of cardiovascular disease and a reduction in life expectancy [3–5].

The estimated global prevalence of PL ranges from 1.7 to 2.8 cases per million inhabitants, including both familial and acquired forms but excluding patients with human immunodeficiency virus (HIV) infection [6]. However, this may be underestimated. Gonzaga et al. [7] reported a higher frequency, identifying 47.3 cases of hereditary lipodystrophies per million inhabitants, with a prevalence of one in every 7.588 inhabitants for autosomal forms of familial partial lipodystrophies (FPLD), based on molecular analyses and electronic medical records. A more recent assessment, combining data from two large international registries and genetic modelling, estimated a prevalence of 19–30 cases per million inhabitants [8]. In Brazil, a multicenter study involving four reference centers examined the clinical features of 424 individuals with FPLD, 106 of whom had genetically confirmed disease [9]. Similarly, a recent nationwide assessment in France, based on medical records from 652 patients, estimated the prevalence of genetically determined lipodystrophy syndromes at 8 per million inhabitants, with 1.6 and 6.4 per million corresponding to generalized and partial forms, respectively [10]. These findings highlight the need for a more precise estimate of disease prevalence in the Brazilian population [7].

Over the past decade, international guidelines from leading clinical teams and medical societies have been published regarding the detection, diagnosis, and treatment of lipodystrophy syndromes [5, 11]. A French protocol was published in 2022 defining criteria for the diagnosis of FLPD type 2 (FPLD2), the most studied form of FPLD [12]. In addition, a more recent systematic review of FPLD2 summarized 113 articles, highlighting the phenotypic characteristics and body composition techniques to help in diagnosis. As a conclusion, they noted that a marked loss of fat in the lower limbs, when associated with other characteristics and noticeable insulin resistance makes the diagnosis of FPLD2 straightforward for trained physicians [13].

Recent studies have documented new variants of FPLD type 3 (FPLD3) and other subtypes [14]. In 2023, a Greek referral center presented a large group with variants in *LMNA* and *PPARG* that differed from those already associated with the disease [15]. The characterisation of variable loss of subcutaneous adipose tissue, in addition to the early metabolic derangements observed in these novel descriptions, has clearly demonstrated the necessity for more precise diagnostic criteria for the recognition of different forms of FPLD [16]. Despite recent advances in knowledge about genetic and phenotypic characteristics, the diagnosis of FPLD remains undervalued or delayed, leading to the development of established clinical complications [17, 18]. In Brazil, the high heterogeneity in phenotypic expression, limited awareness, and absence of established diagnostic criteria for FPLD may contribute to this issue [8, 9, 19].

Several factors motivated the development of this consensus. Although international guidelines for the diagnosis of lipodystrophy syndromes [5, 11] have been adopted in Brazil, they may not be fully applicable to the country’s unique and ethnically diverse population [19, 20]. From a body composition perspective, a continuum exists between individuals with truncal obesity, lipodystrophy-like phenotypes, and FPLD subtypes. Distinguishing between the latter two is important for clinical practice and policy-related purposes [8]. Furthermore, the estimated high prevalence of FPLD in Brazil, combined with limited healthcare resources and a shortage of specialized medical centers, poses significant challenges in the diagnosis and treatment of lipodystrophy-related conditions. Finally, defining a systematic framework for the therapeutic management of lipodystrophy-related conditions is crucial because access to specific therapies for PL is currently restricted.

This expert consensus aimed to: (1) establish clinical criteria for the suspicion and diagnosis of FPLD so that it can be identified by physicians who are not specialists in lipodystrophy; (2) develop a structured approach for clinical and laboratory evaluation, focusing on the investigation of conditions associated with FPLD; and (3) propose a framework for the therapeutic management of FPLD-related disorders and their complications.

## Methods

This expert consensus was developed through discussions involving 15 specialists from lipodystrophy referral centers across Brazil, all of whom are members of the Brazilian Group for the Study of Inherited and Acquired Lipodystrophies (BRAZLIPO), a national collaborative network dedicated to the diagnosis, clinical management, and research of partial and generalized forms of lipodystrophy (www.brazlipo.org).

To support the development of this document, a comprehensive review of the English-language literature was conducted, including articles published up to April 2025. The search strategy included the terms “partial lipodystrophy,” “Köbberling lipodystrophy,” “Dunnigan syndrome,” and “FPLD,” along with other relevant keywords related to each specific topic discussed.

Consensus was achieved through a series of face-to-face meetings supplemented by online and offline activities. Throughout these discussions, the experts addressed five key aspects concerning the management of FPLD: (1) defining the clinical criteria for suspecting the condition; (2) proposing diagnostic criteria to confirm FPLD; (3) proposing a classification system for the most common subtypes based on current medical literature; (4) examining the multisystem manifestations of the disease and establishing guidelines for screening related complications; and (5) developing therapeutic strategies aimed at managing FPLD and its associated metabolic and systemic disorders.

After discussing each topic, the experts reviewed the entire document to ensure consistency and accuracy. The final version of the text was discussed and approved by all participants.

## Results

### Clinical suspicion of FPLD

Clinical suspicion is essential for the timely diagnosis of FPLD. The condition should be suspected in individuals presenting with a reduction or absence of subcutaneous adipose tissue (lipoatrophy) in the limbs (essential criterion), accompanied by at least one of the following conditions commonly associated with FPLD and insulin resistance [9, 16, 17, 21]: acanthosis nigricans, polycystic ovary syndrome (PCOS), hypertriglyceridemia (> 150 mg/dL), and/or low high-density lipoprotein (HDL)-cholesterol (HDL < 50 mg/dL in women or < 40 mg/dL in men), MASLD [22], diabetes mellitus, or impaired fasting glucose [23], or glucose intolerance (particularly if occurring before the age of 40 years), and early coronary atherosclerotic disease (CAD) (diagnosed before the age of 45 years).

If these clinical manifestations occur after puberty, particularly before adulthood, FPLD should be suspected [13]. The criteria for suspecting FPLD are summarized in Table 1.

**Table 1:** Criteria for suspecting familial partial lipodystrophy [3, 5, 8, 11]

Lipodystrophic phenotype (mandatory criterion) Reduction or deficiency of subcutaneous adipose tissue (lipoatrophy) in the lower limbs, with or without accumulation of fatty tissue in the trunk, face, supraclavicular region, and pubic area	
Main conditions related to FPLD (at least one of the following): A. Hypertriglyceridaemia and/or low high-density lipoprotein (HDL)-cholesterol (triglycerides > 150 mg/dL and/or HDL < 50 mg/dL in women or < 40 mg/dL in men) B. Diabetes mellitus, impaired fasting glucose, or glucose intolerance (particularly if occurring before the age of 40 years) C. Metabolic-associated steatosis liver disease (MASLD) D. Early coronary atherosclerotic disease (CAD) (diagnosed before 45 years of age) E. Acanthosis nigricans (or other clinical signs of insulin resistance) F. Polycystic ovary syndrome (PCOS)	

### Diagnosis of FPLD

#### Medical history

Gathering a detailed medical history is essential for diagnosing FPLD. It is important to determine the age at which lipodystrophy and any associated diseases occur. Fat redistribution typically becomes apparent after puberty or in early adulthood, with a more pronounced clinical presentation in women [13, 24], who are often the index cases. Additionally, a history of pre-existing autoimmune diseases [25] or triggering events leading to the loss of fat tissue should be assessed, particularly for differential diagnosis in cases of acquired PL. Given the autosomal dominant inheritance of FPLD, evaluating first-degree relatives for similar appearance and/or common conditions (Table 1) increases the likelihood of diagnosis. Establishing a family pedigree is crucial in suspected cases.

#### Physical examination

The most prominent physical finding in FPLD is the reduction or absence of subcutaneous adipose tissue (lipoatrophy) in the limbs, with or without fat deposition in the trunk, neck, and supraclavicular regions. Despite a rounded face and hyperphagia, individuals with FPLD typically have a disproportionately normal body mass index (BMI) when compared to individuals with obesity who present with similar metabolic complications [26]. Therefore, a BMI < 30 kg/m^2^ and a waist-to-hip ratio (WHR) > 0.85 in females and > 0.95 in males, in conjunction with metabolic abnormalities, increases the likelihood of diagnosis.

Anthropometric assessment should include measurements of circumference (neck, abdomen, and hip) and skinfold thickness (anterior thigh, subscapular, triceps, and calf). A thigh skinfold thickness of < 22 mm in women and 10 mm in men has been proposed as a key diagnostic criterion for partial lipodystrophy [11]. Recent data from a Brazilian study evaluating 37 women with FPLD and 74 healthy controls suggested an anterior thigh skinfold cutoff of 20 mm as the most accurate threshold for identifying FPLD in females [20]. The Köb index, calculated as the ratio of subscapularis to calf skinfold thickness, may also aid in diagnosing FPLD type 1 (FPLD1), with a diagnostic threshold of > 3.477 [27].

In women with FPLD2, characteristic features may include broader shoulders relative to the hips, a height-to-leg-length ratio > 2, and small, widened hands with tapered, infiltrated fingers ("sausage fingers") [12]. Apparent muscularity and prominent veins in both the upper and lower limbs, irrespective of resistance exercise, along with fat accumulation in the pubic mound and genital regions (Dunnigan sign), as well as in the neck, submandibular area (double chin sign), and supraclavicular fossa, are highly suggestive of FPLD2 [28]. Less commonly, Achilles tendon contracture and muscle weakness involving the shoulder and pelvic girdle muscles can be observed [29, 30].

#### Complementary assessment

*Laboratory assessment*

The diagnostic evaluation of FPLD should include a comprehensive laboratory workup to characterize the metabolic profile and exclude secondary causes. A standard lipid panel is essential and includes total cholesterol, HDL-cholesterol, non-HDL cholesterol and triglyceride levels. If available, apolipoprotein B may offer additional insights into atherogenic risk. Screening for diabetes and prediabetes should be performed using fasting glucose and HbA1c levels, and oral glucose tolerance testing may be indicated in individuals with normal fasting glucose levels but with a high clinical suspicion.

Additional assessments should include a complete blood count and platelet count, as well as liver function tests, such as AST (aspartate aminotransferase), ALT (alanine aminotransferase), and gamma-glutamyl transferase, to support fibrosis risk stratification using tools such as the Fibrosis-4 (FIB-4) index. Creatine phosphokinase (CPK) levels should be measured in patients with suspected myopathy, particularly in those with *LMNA* variants.

In women with clinical signs of PCOS, androgen profiling is recommended, including the measurement of total testosterone, androstenedione, dehydroepiandrosterone sulfate (DHEAS), and 17-hydroxyprogesterone levels.

Targeted evaluations are advised to rule out acquired forms of lipodystrophy and other endocrine disorders, particularly in index cases lacking a family history. These tests include HIV serology and complement testing (C3, C4, and CH50) to rule out autoimmune forms. For patients with features of Cushing’s syndrome, a late-night salivary cortisol or 1 mg overnight dexamethasone suppression test should be performed. If acromegaly is suspected, insulin-like growth factor 1 (IGF-1) levels should be measured.

Leptin levels in FPLD can be low or inappropriately normal, which may increase suspicion but does not definitively confirm the diagnosis [31]. Adiponectin levels, which are inversely correlated with triglyceride levels and directly correlated with HDL-cholesterol levels, tend to be low and may contribute to the adverse metabolic profile of affected patients [31, 32].

*Body composition assessment*

A body composition assessment by dual-energy X-ray absorptiometry (DXA) can be used to estimate the percentage of fat and lean mass across the whole body and its segments. In lipodystrophy, key measurements include the proportion (%) of adiposity in the lower limbs, fat accumulation in the genital area, and trunk. An objective method for quantifying lipoatrophy is the fat mass ratio (FMR), which was originally described in patients with HIV-related lipodystrophy. The FMR represents the ratio of trunk to lower limb fat percentage [33]. A Brazilian study comparing DXA measurements of 18 patients with FPLD and 16 controls suggested an FMR cutoff of 1.2 for identifying women with FPLD [34]. Recently, for men, a less-studied group in the context of PL, an FMR cutoff of 1.7 has been proposed, corresponding to the 87 th percentile of the UK Biobank distribution [35].

DXA has also been employed using "fat shadows,” a shading method for assessing body composition in lipodystrophies. This technique has demonstrated high accuracy in identifying congenital generalized lipodystrophy (100% sensitivity and specificity) and partial lipodystrophy (85% sensitivity and 96% specificity) [36]. Using DXA, the percentage of fat in the lower limbs within the 1st percentile (p1) was described as a reliable diagnostic parameter for 50 women and 6 men with FPLD2 when compared to a control group [37]. At last, the French protocol proposed a FMR > 1.2 and/or lower limbs fat mass < 25% of total fat mass as useful indicators for the diagnosis of FPLD2 in women [12].

#### Diagnostic criteria for FPLD

Despite their broad acceptance since the initial proposals, the absence of standardized and universally validated diagnostic criteria for FPLD continues to pose challenges in clinical practice, particularly when dealing with atypical phenotypes of this heterogeneous disorder [11, 12, 19]. In the case of other complex diseases, such as systemic lupus erythematosus and rheumatoid arthritis, diagnostic models based on a combination of clinical and complementary findings have been widely adopted. This rationale has also been applied in clinical trials evaluating therapies for lipodystrophy, including the clinical trial of volanesorsen for severe hypertriglyceridemia [38].

Following this approach, the present expert consensus proposes that the diagnosis of FPLD should be established when there is evidence of peripheral fat loss (mandatory criterion) combined with additional supporting features. Specifically, a diagnosis may be confirmed if the patient meets one of the following criteria (Table 2):

- 1 Mandatory criterion + 2 major criteria,
- 1 Mandatory criterion + 1 major criterion + 2 minor criteria; or,
- 1 Mandatory criterion + a positive genetic test identifying a pathogenic variant associated with a known subtype of FPLD.

**Table 2:** Diagnostic criteria for familial partial lipodystrophy [3, 5, 8, 11]

Criteria and description		
**Mandatory**	**Major**	**Minor**
Documented fat scarcity by: A. Skinfold measurement on the thigh (≤ 10 mm for men and ≤ 20 mm for women) [11, 20] OR B. Dual-energy X-ray absorptiometry (DXA) with a fat mass ratio (FMR) > 1.2 [34] OR C. Lower limbs fat mass < 25% of total body fat (also via DXA), after exclusion of other causes [12]	A. First-degree relative with a documented diagnosis of familial partial lipodystrophy (via genetic or clinical diagnosis) B. Severe hypertriglyceridemia (≥ 500 mg/dL) or acute pancreatitis due to hypertriglyceridemia C. Diabetes, impaired fasting glucose, or glucose intolerance D. Metabolic dysfunction-associated steatotic liver disease	*Clinical history* A. Family history of acute pancreatitis secondary to hypertriglyceridemia B. Polycystic ovary syndrome C. First-degree family history of diabetes mellitus or hypertriglyceridemia (diagnosed before age 40) D. Personal or family history of early coronary artery disease (in the patient or first-degree relative) E. Systemic hypertension diagnosed before age 40 *Laboratory tests* A. Triglycerides between 150 and 499 mg/dL and/or high-density lipoprotein < 50 mg/dL (women) or < 40 mg/dL (men), B. Hypoleptinemia (< 8 ng/mL in men and < 12 ng/mL in women) [83, 90] *Physical examination* A. Normal body mass index or overweight (BMI < 30 kg/m^2^) B. Waist-to-hip ratio > 0.85 (women) and > 0.95 (men) C. Acanthosis nigricans D. Prominent muscularity of the limbs E. Fat deposition in the suprapubic, cervical, and/or submandibular regions (double chin sign) and filling of the supraclavicular área

#### Genetic testing

*When and which genetic test should be performed?*

Genetic testing is useful for confirming clinical diagnoses. In clinical practice, next-generation sequencing (NGS) panels should include genes associated with congenital lipodystrophies, such as *AGPAT2, AKT2, ADRA2 A, BSCL2, CAV1, CAVIN1, CIDEC, CTRC, LIPE, LMF1, LMNA, MFN2, PCYT1 A, PLIN1, POLD1, PPARG,* and *ZMPSTE24*. Among these, pathogenic variants in *LMNA, PPARG, AKT2, PLIN1, CAV1, MFN2, CIDEC, LIPE, POLD1,* and *ZMPSTE24* are also associated with known FPLD. Additionally, other genes, such as *ABCA1, APOA5, APOC2, CFTR, CYP27 A1, GPIHBP1, LIPA, LMNB2, LPL, PRSS1, PSMB8, SMPD1,* and *SPINK1*, while not directly linked to recognized FPLD subtypes, could be included in extended genetic panels due to their biological relevance. If an identified variant has not been described in the literature, confirming a new pathogenic variant is crucial using Sanger sequencing based on capillary electrophoresis [39].

Genetic testing for FPLD is useful in the following situations:

1. Individuals who met the suspected criteria for FPLD (Table 1).
2. Individuals who do not meet the suspected or diagnostic criteria (Tables 1 and 2) but have family members who have been clinically or genetically diagnosed with FPLD (cascade screening).

In the first case, genetic evaluation serves as a diagnostic test, whereas in the second case, it functions as a predictive test. Since FPLDs exhibit significant phenotypic and genetic heterogeneity, this investigation is useful for screening family members and helps to understand the natural history of the disease, treatment planning, and genetic counselling.

*How to interpret NGS results?*

Negative genetic test with clinical criteria for diagnosing FPLD: In this scenario, the patient may carry a variant of unknown significance that should be considered in the diagnosis of FPLD1 or FPLD type X (FPLD-X). The index patient should be monitored for lipodystrophy-related disorders, and family members should be screened for clinical criteria to determine the disease inheritance pattern.

Positive genetic test with clinical criteria for diagnosing FPLD: The diagnosis of FPLD is confirmed. If genetic tests are available, the impact of the identified variant and its presence in other family members should be investigated further.

Positive genetic test without clinical criteria for diagnosing FPLD: Clinical evaluation and follow-up is recommended to monitor FPLD-related metabolic conditions and assess the pathogenicity of the variant, inheritance pattern, and genotype–phenotype relationship. Notably, changes in body composition were described before puberty, particularly in girls with FPLD2 [40]. In light of this, screening for FPLD should be considered at the age of ten in girls from families with pathogenic variants of *LMNA* to anticipate metabolic complications [41, 42].

*What about genetic counselling?*

Genetic counselling is essential for patients with FPLD, helping them to understand how heredity contributes to the onset of the disease and the likelihood of its occurrence in their offspring and other family members. In addition, it facilitates decision-making processes related to family planning and the available treatments.

### Classification of FPLD subtypes

Currently, 11 forms of FPLD have been classified based on the patient's clinical characteristics, related genes, and/or associated pathogenic variants (Table 3). According to the Online Mendelian Inheritance in Man (OMIM) database, six forms follow an autosomal dominant inheritance pattern (types 2 [LMNA], 3 [PPARG], 4 [PLIN1], 7 [CAV1]), AKT2-related FPLD, and ADRA2-related FPLD), whereas three follow an autosomal recessive inheritance pattern (types 5 [CIDEC], 6 [LIPE], and FPLD with MFN2 associated lipomatosis) [32]. An exception is the Köbberling type (Type 1), for which no genetic mutations have been identified [11, 17].

**Table 3:** Classification of FPLD based on genotype and/or phenotype

FPLD type (#OMIM)	Genetic variant (*OMIM) and inherited pattern	Affected protein and main function	Phenotype	Time of onset and pattern of fat distribution
FPLD 1 (Köbberling syndrome)	Polygenic		Kob index > 3.47, truncal obesity, lipoatrophy of the limbs, “ledge” between normal and lipodystrophic areas [43, 27]	Adulthood
FPLD 2 (Dunnigan syndrome) (#151660)	*LMNA *(*150330) AD	Lamin-A/C	Double chin with fat depots in the face, neck, supraclavicular regions, and genitalia; muscular appearance in the limbs; phlebomegaly [2, 18, 47]	Childhood in females, early adulthood in males
FPLD 3 (#604367)	*PPARG *(*601487) AD	PPARG (adipogenesis)	Distal lipoatrophy, insulin-resistant diabetes with severe hypertriglyceridemia [16, 49]	Childhood and early adulthood
FPLD 4 (#613877)	*PLIN1 *(*170290) AD	Perilipin-1 (lipid storage in droplets)	Lower limb lipoatrophy (anti-perilipin antibodies) [48]	Childhood or adulthood
FPLD 5 (#615238)	*CIDEC *(*612120) AR	CIDEC-3 (lipid droplet fusion)	Loss of subcutaneous fat from the limbs, diabetes mellitus with insulin resistance [39, 87]	Childhood
FPLD 6 (LIPE-associated lipomatosis) (#615980)	*LIPE *(*151750) AR	Hormone-sensitive lipase (HSL)	Distal lipoatrophy with multiple symmetric lipomatosis, insulin-resistant diabetes, and progressive myopathy [39, 87]	Adulthood
FPLD with MFN-associated lipomatosis (#151800)	*MFN2 *(*608507) AR	Mitofusin-2 (mitochondrial membrane fusion)	Lipoatrophy of the limbs with multiple symmetric lipomatosis, polyneuropathy, insulin resistance, and low levels of leptin and adiponectin [39, 87]	Early adulthood
FPLD 7 (#606721)	*CAV1 *(*601047) AD	Caveolin-1 (lipid droplet formation)		
*AKT2-related FPLD*	*AKT2 *(*164731) AD	AKT2 (insulin receptor signalling)	Loss of subcutaneous fat from the limbs [39, 87]	Adulthood
ADRA2-related FPLD	*ADRA2A*	Adrenoreceptor α2A (Lipolysis)	Fat loss in trunk and extremities and increase of fat in face and neck [91]	Adulthood
FPLD type X	*ND*	ND	Kob index < 3.47, lipoatrophy of the limbs, insulin-resistant diabetes with severe hypertriglyceridemia	Early adulthood

*OMIM* Online Mendelian Inheritance in Man (https://www.omim.org/), *AR* autosomal recessive, *AD* autosomal dominant, *ND* not determined

#### FPLD type 1: Köbberling syndrome

FPLD1 is characterized by the loss of subcutaneous fat restricted to the gluteal region and extremities, as well as excessive accumulation in the trunk area, with a normal distribution in the face and genitalia [43]. In patients with FPLD1, complications such as hypertriglyceridemia, pancreatitis, and coronary artery disease seem more prevalent than in those with FPLD2. Additionally, leptin levels in FPLD1 correlate more directly with BMI [27, 43].

A comprehensive analysis of the human genome has suggested a polygenic origin for FPLD1 [44]. In a genome-wide association study, Udler et al. identified five major loci groups in patients with type 2 diabetes (T2D), with one displaying a “lipodystrophy-like” fat distribution [45]. This suggests that some clinically diagnosed patients with T2D may actually belong to this “lipodystrophy-like” cluster, highlighting the likelihood of underdiagnosis by diabetologists and other medical specialists [8, 35, 44–47]. Given the vast heterogeneity in the presentation of FPLD without known pathogenic variants, this expert consensus recommends that individuals meeting this profile and with a Kob index > 3.477 must be classified as FPLD1. Those with FPLD features but not meeting this criterion should be classified as FPLD type X [36].

#### FPLD type 2: Dunnigan syndrome

Pathogenic variants in *LMNA* are the most frequent among FPLDs with known genetic alterations, accounting for approximately 80% of reported cases [2, 18]. Most patients with FPLD2 have a heterozygous variant that replaces the basic amino acid at position 482 (arginine) with a neutral one [28]. Laminopathies are clinically diverse and can potentially cause muscular dystrophy, neuropathy, cardiomyopathy, progeroid syndromes, and lipodystrophy [47].

Patients with Dunnigan syndrome may present with a Cushingoid appearance, loss of subcutaneous fat in the limbs, and variable accumulation of fat in the trunk, suprapubic, supraclavicular, and cervical regions of the body. Additionally, prominent musculaturity and phlebomegaly in the arms and legs help to distinguish FPLD2 from other partial forms and facilitate the recognition of the FPLD2 phenotype, particularly in women. However, the higher prevalence in females should not be solely attributed to their appearance. In males, phenotypic expression is associated with a less aggressive metabolic profile, and diagnosis often occurs later in life [28, 48].

#### FPLD type 3 (FPLD3)

Pathogenic variants in peroxisome proliferator-activated receptor gamma (*PPARG)*: Known for its role in adipogenesis, *PPARG* regulates other genes involved in adipocyte differentiation, maintenance, lipogenesis, and fatty acid transport [16]. In individuals with FPLD3, lipoatrophy in the limbs tends to be less pronounced than that in FPLD2, whereas metabolic alterations are more severe [16, 49].

A recent study investigated the clinical features of 41 *PPARG* pathogenic variants in 91 patients with FPLD3. Females were the most diagnosed (75.8%), with an average age of 20 years at the onset of FPLD. The most common metabolic conditions related to FPLD3 were hypertriglyceridemia (91%), MASLD (87.5%), diabetes (77%), hypertension (59.5%), and PCOS (58.7%) [14].

Another study comparing the phenotypic differences between 256 patients with FPLD2 and 32 patients with FPLD3 found that type 3 patients had a significantly higher prevalence of hypertriglyceridemia (84% vs. 66%) and diabetes (72% vs. 44%), as well as increased skinfolds and regional body fat compared with individuals with FPLD2 [16].

The suggested classification of familial partial forms based on the genotype–phenotype correlations is shown in Table 3.

The proposed diagnostic criteria were applied to a sample from a previously studied population for validation [9]. In a sub-analysis involving 94 patients with known pathogenic variants linked to PL, confirmed through molecular analysis across four centers, 67 patients (71.3%) satisfied the diagnostic criteria by exhibiting the mandatory criterion in conjunction with two major criteria. In a sub-analysis involving 28 patients from two lipodystrophy-specialized centers, all participants exhibited a major criterion in conjunction with two minor criteria. The distribution of minor criteria was as follows: one patient (3.57%) had a total of 3 criteria, two patients (7.14%) had 4, one patient (3.57%) had 5, three patients (10.71%) had 6, six patients (21.43%) had 7, five patients (17.86%) had 8, eight patients (28.57%) had 9, and two patients (7.14%) had a total of 10 criteria. In the genetic evaluation of this subgroup comprising 28 patients, the distribution of gene variants was as follows: variants in the *LMNA* gene were identified in 19 patients (67.8%), *PPARG* in five (17.9%), *MFN2* in one (3.6%), *SPINK1* in one (3.6%), and *BSCL2* in one (3.6%). Additionally, one patient (3.6%) presented a variant of the *AKT* gene in association with an *LMNA* variant. Notably, one of the major criteria, having a first-degree relative with a documented diagnosis of familial partial lipodystrophy (via genetic or clinical diagnosis), was not used in this proof-of-concept analysis.

**Fig. 1:**
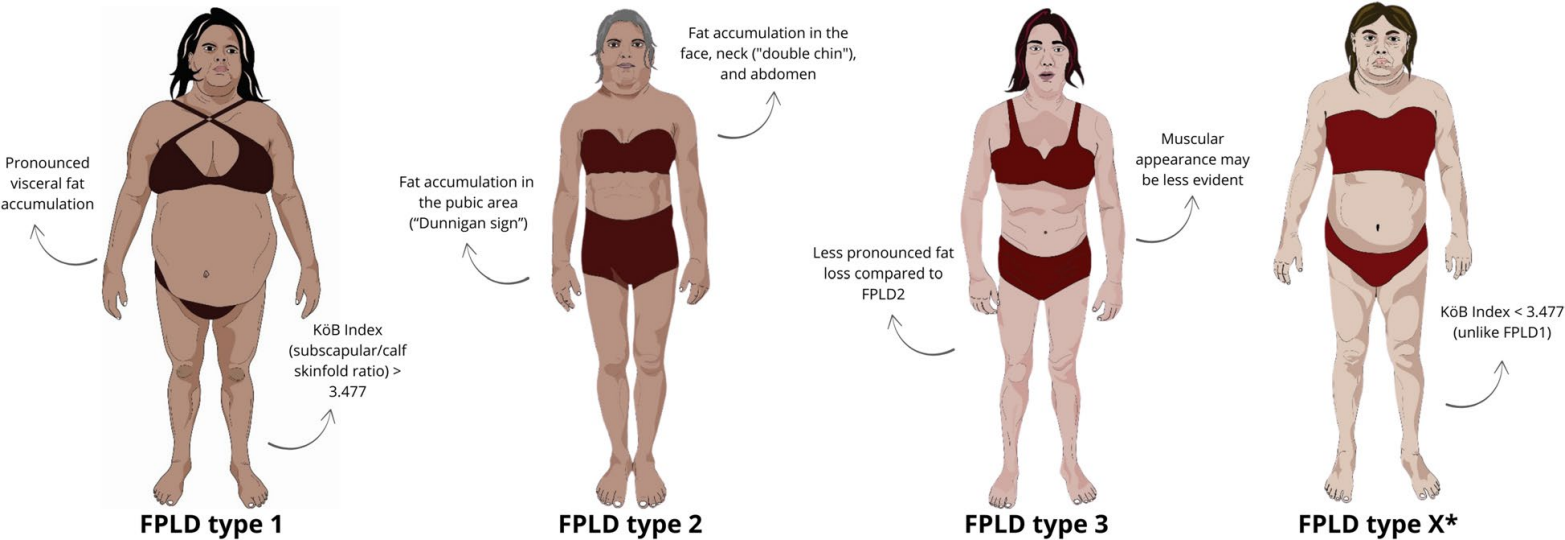
Visual spectrum of familial partial lipodystrophy (FPLD) phenotypes and their specific hallmarks. *FPLD type X includes patients with lipodystrophic features, no identified pathogenic variant, and a KöB index < 3.477, differentiating it from FPLD1

**Fig. 2:**
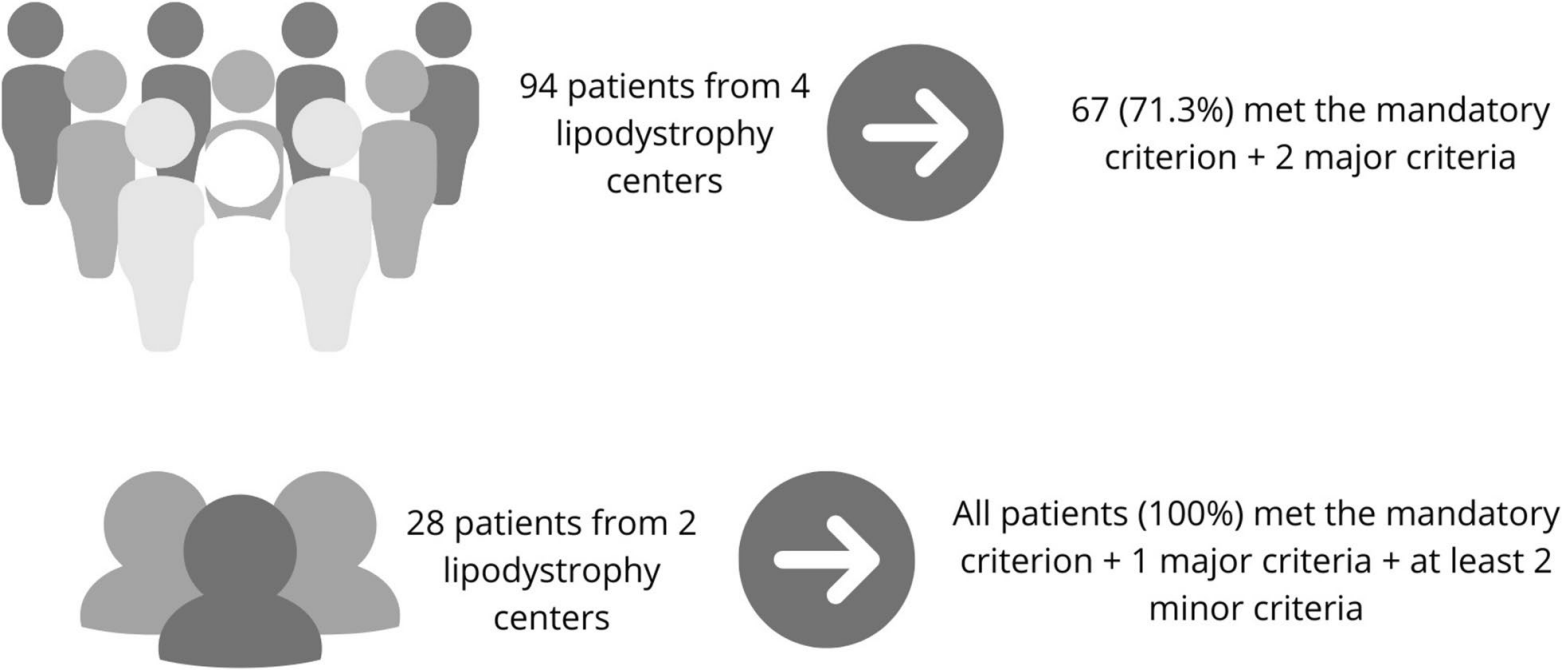
Performance of the proposed diagnostic criteria in genetically confirmed FPLD cases from the BRAZLIPO registry

Figure 1 illustrates the lipodystrophy axis in Brazil, covering the spectrum of FPLD phenotypes. Figure 2 shows the performance of the proposed diagnostic criteria in genetically confirmed FPLD cases from the BRAZLIPO registry.

### Screening for multisystem manifestations and FPLD-related complications

FPLD is usually diagnosed after complications have developed, reinforcing the importance of early detection. Notably, the severity of many complications observed in patients with FPLD, particularly cardiovascular and renal outcomes, may be determined by a direct relationship with lipodystrophy and the duration of inadequate control of associated conditions, such as diabetes, high blood pressure, or dyslipidemia. To differentiate between these scenarios, determining the natural history of the disease and its various clinical forms is essential, in addition to appropriate screening and early management of the complications. The main clinical manifestations and complications associated with FPLD are shown in Table 4.

**Table 4:** Multisystem clinical manifestations associated with FPLD [1, 5, 12, 29, 30, 68–70]

Manifestations	Clinical findings
Dermatological	Skin thickening, seborrhoea, acne, lipomas, acanthosis nigricans, leuko-melanoderma, scleroderma-like syndrome
Endocrine and metabolic	Dyslipidemia (hypertriglyceridemia and low high-density lipoprotein), dysglycemia (glucose intolerance, impaired fasting glucose, or diabetes mellitus [DM]), polycystic ovary syndrome (hyperandrogenism, oligomenorrhea, infertility), hyperphagia
Cardiovascular	Systemic arterial hypertension (SAH), silent myocardial ischaemia, dilated cardiomyopathy, and arrhythmias
Gastrointestinal	MASLD with potential progression to cirrhosis, acute pancreatitis due to hypertriglyceridemia
Genitourinary	Chronic kidney disease (secondary to DM and SAH), nephropathy with proteinuria
Neurological	Diabetic or laminopathy-related neuropathy, cerebrovascular disease
Musculoskeletal	Muscle hypertrophy or pseudohypertrophy, myalgia or myopathy (FPLD2), chronic and diffuse pain
Psychiatric	Depression, anxiety, and impaired quality of life

#### Endocrine and metabolic manifestations

Dyslipidemia and diabetes are the most common metabolic conditions. A recent systematic review evaluating 494 individuals with FPLD due to *LMNA* variants reported a prevalence of dyslipidemia and diabetes of 83% and 61%, respectively [50]. Similarly, a retrospective study in the Brazilian population found that among the 424 evaluated patients (25% with positive pathogenic variants), diabetes and severe hypertriglyceridemia were present in 57.5% and 35% of patients, respectively [9].

Atherogenic dyslipidemia, characterized by hypertriglyceridemia and low HDL-c levels, is a typical finding in patients with FPLD [51]. Hypertriglyceridemia is one of the earliest metabolic manifestations in the natural history of the disease, preceding the onset of hyperglycemia, and may progress to severe hypertriglyceridemia (triglyceride ≥ 500 mg/dL) in some cases [31, 52].

A study of 102 adult patients with FPLD2 found that 65% had either diabetes (41%) or prediabetes (24%), despite a relatively young average age (39 years). Notably, among those with prediabetes, 86% exhibited 2-h glucose intolerance (with normal fasting glucose levels) [53].

#### Cardiovascular manifestations

Atherogenic dyslipidemia is common in patients with FPLD and is a major contributor to increased cardiovascular risk and reduced life expectancy [54]. A retrospective analysis of 258 patients with FPLD2 (195 women and 63 men) revealed a higher prevalence of hypertriglyceridemia and diabetes in women than in men. In addition, the rate of cardiovascular events was similar in men and premenopausal women. Additionally, Guidorizzi et al. reported a 10.4% prevalence of established atherosclerotic disease in a retrospective study of 424 Brazilian patients with FPLD [9]. These findings indicate an increased cardiovascular risk and the necessity of early screening for subclinical atherosclerosis in these patients [51].

Another key concern among individuals with FPLD2 is the assessment of the heart rhythm. A case series of patients with FPLD2 with the R482W variant identified myopathies, muscular dystrophies, dilated cardiomyopathy, and electrical conduction defects [55]. Similarly, a multicenter study by Eldin et al. involving 122 patients showed a higher prevalence of arrhythmias (odds ratio: 3.77, 95% confidence interval: 1.45–9.83) in patients with *LMNA* variants (particularly non-482 codons) [56]. Thus, this expert consensus recommends that when FPLD is diagnosed, resting electrocardiogram and echocardiogram should be performed to assess for structural changes (hypertrophic or dilated cardiomyopathy). Another parameter to be evaluated is epicardial fat, an emerging marker of cardiovascular risk, whose thickness is increased and inversely proportional to leptin levels in individuals with FPLD2 [57].

Cardiac monitoring with a Holter monitor may be considered at the clinician's discretion to evaluate conduction defects (non-482 codon *LMNA* variants). Additionally, screening for subclinical atherosclerotic disease using functional provocative testing (stress test, myocardial scintigraphy, and/or stress echocardiogram) may be considered. In selected cases, coronary computed tomography angiography with calcium scoring may also be used to investigate silent myocardial ischemia.

#### Hepatic manifestations

The full spectrum of MASLD, including hepatic steatosis, metabolic dysfunction-associated steatohepatitis (MASH), fibrosis, cirrhosis, and hepatocellular carcinoma, has been described in patients with lipodystrophy [58]. Some authors suggest that MASLD in lean individuals should raise suspicion of lipodystrophy, as it is one of the most frequent findings in over 90% of patients with FPLD [4, 18, 58].

Ajluni et al. investigated the severity of MASLD in 23 individuals with partial lipodystrophy (seven with FPLD2) using Dixon magnetic resonance imaging (MRI) and liver biopsy. Their findings showed a high prevalence of MASH with fibrosis, as 22 patients met the criteria for MASH (mean NAS [NAFLD Activity Score] = 6 ± 2), and 18 patients (78.3%) demonstrated some degree of fibrosis in the histopathological examination. They also found a positive correlation between liver fat quantified by MRI and HbA1c and log-transformed triglyceride levels [4].

Another study using transient hepatic elastography (THE) in 32 young women with FPLD (15 of whom had FPLD2; mean age = 49.1 ± 11.7 years; BMI = 25.5 kg/m^2^ [19.9–39.1]) found a positive correlation between abdominal circumference and measures of hepatic steatosis and fibrosis evaluated by THE. The same analysis demonstrated a direct association between triglyceride levels and hepatic steatosis, reinforcing the role of excess visceral fat in MASLD severity in this group of patients with FPLD [59].

According to the Brazilian guideline for the management of MASLD in people with prediabetes or T2DM, evaluation with elastography is recommended for the screening of liver fibrosis when the FIB-4 index is ≥ 1.3 [22]. Given the early onset and severity of hepatic manifestations in the previously described case series, it is likely that the fibrosis risk estimated based on isolated FIB-4 score calculations is underestimated when assessing MASH in patients with FPLD. Therefore, this expert consensus suggests that upon FPLD diagnosis, hepatic ultrasonography combined with shear wave elastography or transient elastography should be the first-line examination to rule out liver fibrosis. MRI elastography should be considered in cases of conflicting results, when concurrent causes are suspected, or to further rule out the risk of advanced liver fibrosis.

#### Pancreatic manifestations

A study involving 11 female patients with FPLD2 used Dixon MRI to assess pancreatic and hepatic fat, revealing an increased pancreatic fat content in the FPLD2 population compared with that in controls (5.26 ± 1.5 vs. 4.08 ± 0.64, p = 0.034). Additionally, pancreatic fat was found to be inversely related to beta cell function, as assessed using the disposition index [60].

Acute pancreatitis secondary to hypertriglyceridemia is one of the most serious complications of partial lipodystrophy. An analysis of 74 patients with FPLD2 showed that the prevalence of severe hypertriglyceridemia and acute pancreatitis was higher in patients diagnosed with T2D than in those without (14.3% and 10.7%, respectively) [61].

Another recent systematic review with a retrospective evaluation of 494 individuals with *LMNA*-related lipodystrophy showed that 7.8% (n = 39) had experienced at least one episode of acute pancreatitis, with a 3.2-fold higher risk in those with diabetes and a 12-fold higher risk in those with hypertriglyceridemia [50]. In another study, pancreatitis was observed in 8.5% of patients, with a higher prevalence of subtype 3 [9].

#### Renal manifestations

Chronic kidney disease and proteinuria have been reported in patients with FPLD, with the prevalence of severely increased albuminuria (≥ 300 mg/g) reaching approximately 21%. Biopsy findings in these individuals have revealed glomerular hypertrophy, mesangial expansion, podocyte injury, diabetic renal disease, focal segmental glomerulosclerosis, and membranoproliferative glomerulonephritis [47]. Reports on patients with FPLD2 and proteinuric kidney disease suggest the occurrence of this association in *LMNA* variant carriers [62]. However, the most commonly observed renal involvement is secondary to poorly controlled hypertension and diabetes [63].

#### Reproductive system manifestations

In women with FPLD, PCOS is a common clinical manifestation expected due to severe insulin resistance. However, this resistance is partial, meaning that while specific tissues exhibit high resistance, others, such as the ovaries, do not [64]. In a study of 14 women with FPLD2, the prevalence of PCOS exceeded 50%, and infertility was observed in approximately 30% of cases [65]. Gambineri et al. identified 18 cases with a partial lipodystrophy phenotype among 1200 patients attending a clinical center for complaints related to PCOS. Of these, 12 cases were diagnosed with familial forms, accounting for 1% of the population; nine cases had variants in the *LMNA* gene, one in the *PPARG* gene, and two in the *PLIN1* gene [66].

A retrospective study by Valerio et al. involving eight patients with FPLD2 found a 50% prevalence of PCOS. Among these patients, the rate of obstetric complications was high: 37.5% experienced spontaneous abortions, 25% developed gestational diabetes, and 50% delivered neonates with macrosomia [67]. Additionally, obstetric complications were more frequent than those in the general population, with rates of 30% for gestational diabetes, 50% for spontaneous abortions, and just over 10% for preeclampsia and fetal death.

Given the increased frequency of maternal–fetal complications compared with the general population, intensive high-risk prenatal care is essential. Additionally, hypertriglyceridemia, which is common in these patients, may worsen during pregnancy, increasing the risk of pancreatitis.

When counseling on contraception, oral contraceptives containing estradiol should be avoided, as they may exacerbate pre-existing metabolic issues such as dyslipidemia [30].

Currently, there are no available data in the medical literature regarding infertility or changes in the reproductive system in men with FPLD.

#### Dermatological manifestations

Hirsutism and acanthosis nigricans in skinfolds and friction-prone areas (axillary, cervical, and inguinal) are among the most common dermatological manifestations of FPLD. The skin is often thick and may exhibit a seborrheic or acne-like appearance. Other dermatological findings may include skin tags and eruptive xanthomas on extensor surfaces. Leukoderma, a syndrome similar to scleroderma with telangiectasias, has also been reported. The presence of lipomas is common, particularly in cases associated with variants of *LMNA, MFN2*, or *LIPE* [30].

#### Neuromuscular and rheumatological manifestations

Myalgias, with muscle weakness or muscular dystrophy, are primarily described in FPLD2 and are associated with the *LMNA* R482W mutation [55]. In patients with FPLD, peripheral neuropathy with characteristics similar to those caused by diabetes is common and can significantly affect the quality of life.

#### Psychological manifestations

FPLD can significantly affect the quality of life (QoL) of patients. A recent patient perspective study on the disease demonstrated its adverse effects on physical, emotional, and social well-being, as well as self-esteem and body image. Additionally, patients reported experiencing discrimination and stigma due to their appearance and health conditions [68, 69].

One study highlighted that pain is a major determinant of QoL and psycho-emotional health in these patients. Patients with moderate-to-severe pain have worse quality-of-life scores, including physical functioning, energy, emotional well-being, social functioning, and general health [70].

### Therapeutic approach

The therapeutic approach for FPLD should prioritize behavioral treatment (lifestyle modifications) and early intervention for conditions associated with FPLD.

#### Behavioural: lifestyle modification

Current dietary recommendations for individuals with FPLD are based on clinical experience and treatment of associated conditions. Caloric restriction can be challenging because disproportionate hunger may occur in some clinical forms owing to low plasma leptin levels.

Current guidelines for the diagnosis and treatment of lipodystrophy recommend a diet containing 50–60% carbohydrates, 20–30% fat, and approximately 20% protein [5]. Abstinence from smoking and alcohol should be strongly encouraged, particularly alcohol abstinence, in patients with moderate to severe hypertriglyceridemia.

Given the clinical heterogeneity of FPLD, nutritional recommendations should be tailored to control metabolic abnormalities. Very low-calorie diets (VLCD) may be beneficial for managing metabolic issues. In a proof-of-concept study, Foss-Freitas et al. demonstrated that a VLCD (800 kcal/day) led to a 40% and 1.4% reduction in triglyceride levels and HbA1c, respectively, after four months in a 38-year-old woman with FPLD2. These findings suggest that periods of more severe caloric restriction may be particularly beneficial for achieving adequate metabolic control in these patients [71].

Following the assessment of structural heart abnormalities, the promotion of aerobic exercise is advisable. It is essential to conduct appropriate screening for subclinical atherosclerotic disease in patients presenting with cardiovascular risk factors. Additionally, the encouragement of resistance exercises is recommended.

#### Management of diabetes

Treatment should be individualized based on hyperglycemia levels, renal function, and drug availability. Metformin is widely recommended as a first-line therapy because it is effective for glycaemic control and the management of associated metabolic complications. In cases of severe insulin resistance, high-dose insulin may be necessary to optimize glycemic control [47].

Thiazolidinediones, which selectively act on *PPARG*, are of particular interest not only for their efficacy in glycemic control but also for their ability to reduce lipotoxicity by lowering free fatty acid levels and improving hepatic steatosis associated with MASLD, thereby promoting healthy adipose tissue expansion [72, 73]. However, these medications may induce fat deposition in areas unaffected by lipodystrophy, which should be considered during treatment planning.

Additionally, elevated levels of dipeptidyl peptidase-4 (DPP-4) have been identified in individuals with FPLD, suggesting that DPP-4 inhibitors may be a relevant therapeutic option for managing diabetes in these patients [74].

GLP-1 receptor agonists (GLP-1RA) have emerged as promising alternatives. A retrospective study investigated their use in 14 patients with FPLD (13 with FPLD type 1 and one with FPLD type 2) and demonstrated efficacy and safety comparable to those of age- and sex-matched individuals with T2D [75]. However, caution should be taken in patients with an increased risk of pancreatitis. Another recent retrospective analysis of 57 patients with FPLD from the French Lipodystrophy Reference Network showed significant improvements in BMI, HbA1c, and triglycerides after one year of GLP-1RA therapy, with one case of acute pancreatitis associated with severe hypertriglyceridemia in a non-compliant patient [76].

Notably, a recent study described impressive results with the use of a dual incretin, tirzepatide, 15 mg, once a week, prescribed as monotherapy for glycemic control in two patients with congenital generalized lipodystrophy type 1 [77]. Another observational cohort study of 17 patients with lipodystrophy, 14 of whom had FPLD, who received tirzepatide clinically, was conducted as part of ongoing natural history studies. After a median 8.7 months of follow-up, significant reductions were observed in BMI (− 1.7; range: − 5.9 to 0.9 kg/m^2^; p = 0.008), HbA1c (− 1.1%; range: − 6.3 to − 0.1%; p < 0.001), triglycerides (− 65 mg/dL [− 0.73 mmol/L]; range: − 3820 to 43 mg/dL [− 43.2 to 0.49 mmol/L]; p = 0.003), and total daily insulin requirements (− 109; range: − 315 to 0 units/day; p = 0.002) [78]. These findings suggest that tirzepatide may be an effective treatment for patients with FPLD, with promising results in terms of metabolic control.

Another therapeutic group for diabetes management is the sodium-glucose cotransporter type 2 (SGLT2) inhibitors. A retrospective review of the medical records (N = 22 for safety and N = 12 for efficacy) of patients with partial lipodystrophy treated with SGLT2 inhibitors (canagliflozin, empagliflozin, and dapagliflozin) showed promising results [79]. After 12 months of treatment, a significant reduction was observed in HbA1c levels (from 9.2 ± 2.0% to 8.4 ± 1.8%; p = 0.028), along with reductions in systolic (p = 0.011) and diastolic (p = 0.013) blood pressures. Although C-peptide levels showed a trend towards reduction (p = 0.071), fasting glucose levels, lipid profiles, and estimated glomerular filtration rates remained unchanged. Reported adverse events included extremity pain, hypoglycemia, diabetic ketoacidosis (in a patient with non-adherence to insulin), pancreatitis (in a patient with a history of the condition), and urogenital fungal infections. Thus, SGLT2 inhibitors may effectively reduce HbA1c levels in patients with partial lipodystrophy, with a safety profile similar to that observed in patients with T2D. However, careful evaluation of the risks and benefits is necessary before including this class in lipodystrophy treatment, and additional studies with larger patient populations are required to confirm these findings.

Although data on bariatric surgery in patients with lipodystrophy are scarce, there is evidence that some individuals who underwent Roux-en-Y gastric bypass, particularly those with pathogenic variants in *LMNA* and *PLIN1*, or without variants in *LMNA* and *PPARG* (suggesting FPLD1), showed significant improvement in metabolic parameters and weight loss. These interventions suggest a potential metabolic benefit in carefully selected patients, highlighting the need for further investigation of surgical and pharmacological therapies in this population [47].

#### Management of dyslipidemia

Dyslipidemia treatment should focus on the primary and secondary prevention of cardiovascular events. After estimating cardiovascular risk-which is elevated in patients with FPLD-the combination of high-potency statins followed by ezetimibe are recommended as the first-line therapy to achieve LDL-cholesterol targets [80, 81].

Secondary treatment targets, such as non-HDL cholesterol and apolipoprotein B, may be used to treat mild or moderate hypertriglyceridemia. Fibrates and long-chain omega-3 fatty acids may be used in cases of severe hypertriglyceridemia (≥ 500 mg/dL) [5].

#### Volanesorsen

The efficacy and safety of volanesorsen, an antisense inhibitor of apolipoprotein C-III, were evaluated in a 52-week phase 2/3 study, which randomized participants in a 1:1 ratio to receive weekly administration of volanesorsen (285 mg) or a placebo. This study demonstrated an 88% reduction in triglyceride levels after three months and a significant reduction in the liver fat fraction in 40 individuals with FPLD [38].

Therefore, the use of volanesorsen 285 mg in weekly subcutaneous injections might be considered in individuals with FPLD and either a history of acute or recurrent pancreatitis, or at high risk of pancreatitis due to severe hypertriglyceridemia (≥ 500 mg/dL) unresponsive to previously described lipid-lowering therapy. Thrombocytopenia is the main side effect described, so that platelet blood count should be monitored at a weekly basis and kept over 140.000/μL.

#### Leptin-based therapy (metreleptin)

Metreleptin, a recombinant human leptin analog, has demonstrated efficacy in reducing hyperphagia and improving insulin resistance and its complications in patients with lipodystrophy [82].

In previous studies, individuals with FPLD who responded well to metreleptin were those with more significant metabolic abnormalities (HbA1c > 8.0% or triglycerides ≥ 500 mg/dL) [83]. Meral et al. compared patients with FPLD2 with hypoleptinemia (serum leptin < 7th percentile of normal) versus those with moderate hypoleptinemia (serum leptin in the 7–20th percentiles) and observed that leptin therapy was equally effective in reducing serum and hepatic triglyceride levels without improving hyperglycemia. No reliable serum leptin cutoff point has been identified for selecting lipodystrophy patients who respond to metreleptin [84].

Although most studies have been conducted on FPLD2, Sekizkardes et al. evaluated the response of seven patients with FPLD3 and compared it with that of 22 patients with FPLD2 [85]. After 12 months of metreleptin use, the mean HbA1c level decreased from 9.2% to 7.7% in the FPLD3 group and from 7.8% to 7.3% in the FPLD2 group, respectively. Triglyceride levels also declined, with an average reduction from 1377 to 680 mg/dL in the FPLD3 group and from 332 to 293 mg/dL in the FPLD2 group, respectively. The study concluded that leptin treatment appears to have similar efficacy in treating metabolic abnormalities in patients with FPLD2 and FPLD3 and confirms better responses in patients with worse metabolic control in both groups. A greater chance of reducing triglycerides by > 30% and HbA1c by > 1% was observed in patients with baseline triglycerides ≥ 500 mg/dL and HbA1c > 8%.

In another recent analysis, among 103 patients with generalized or partial forms treated with metreleptin (20 of whom had FPLD2), a reduction in mortality risk was observed in 65% of patients. This study demonstrated that treated patients had more severe disease than metreleptin-naïve patients [86].

Metreleptin is generally well tolerated, with hypoglycemia being one of the potential side effects of improved metabolic control and the development of anti-metreleptin antibodies. Although antibody development is expected, only a minority of patients develop neutralizing antibodies that reduce the biological activity of leptin and worsen metabolic control [82]. Therefore, the use of metreleptin from 0.05 mg/kg (starting dose) to 10 mg (maximum dose) in daily injections should be considered in individuals with FPLD and inadequate metabolic control (HbA1c > 8.0% and/or triglycerides ≥ 500 mg/dL), despite the use of optimized lipid-lowering and antidiabetic therapies.

## Summary of recommendations

Based on current evidence, this expert consensus provides structured recommendations for the diagnosis, classification, screening, and management of FPLD. The following summary consolidates key recommendations to assist clinicians in diagnosing and optimizing care for individuals with FPLD:

- The effective management of FPLD necessitates early clinical suspicion, comprehensive screening for multisystem complications, and implementation of individualized treatment strategies tailored to the genetic subtype and clinical severity of the condition.
- Clinical suspicion should be heightened in patients exhibiting peripheral lipoatrophy, especially when it is concomitant with metabolic abnormalities, such as hypertriglyceridemia, insulin resistance, MASLD, or early onset cardiovascular disease.
- The diagnostic process relies on clinical criteria, anthropometric assessments, laboratory evaluations, and genetic testing when accessible.
- All patients with FPLD should undergo regular metabolic screening including lipid profiling, glycemic status, and liver function tests.
- Cardiovascular screening with electrocardiogram and echocardiogram is particularly important in patients with *LMNA* pathogenic variants, given the higher risk of dilated cardiomyopathy, arrhythmias, and conduction abnormalities. Holter monitoring may also be considered, particularly for *LMNA* non-482 variants, to detect subclinical rhythmic disturbances. Screening for subclinical atherosclerosis may be individualized based on clinical risk factors.
- Non-invasive techniques, such as transient elastography or magnetic resonance imaging (MRI), should be employed for the assessment of hepatic steatosis and fibrosis, with periodic re-evaluation contingent upon clinical risk factors.
- Central to the treatment strategy are lifestyle interventions that should encompass a personalized hypocaloric diet and a structured exercise regimen that incorporates both aerobic and resistance training. Nutritional modifications must account for the heightened risk of hypertriglyceridemia and the potential for MASLD even in individuals who do not have obesity.
- Metformin continues to be the first agent for glycemic management. Nevertheless, in instances of significant insulin resistance, GLP-1 receptor agonists, SGLT2 inhibitors, or insulin should be considered. Thiazolidinediones may provide particular advantages for patients with FPLD3 owing to their effects on PPARG. Tirzepatide should be considered as an effective treatment for metabolic Aimprovement of diabetes, insulin resistance and related complications. Dyslipidemia should be addressed with high-intensity statins and ezetimibe, whereas fibrates and omega-3 fatty acids are recommended for severe hypertriglyceridemia (≥ 500 mg/dL).
- Advanced therapies may be considered for selected patients. Metreleptin could be an option for individuals with poorly controlled diabetes (HbA1c level > 8.0%) and/or severe hypertriglyceridemia, particularly when conventional therapies fail. Similarly, volanesorsen may be considered in cases of severe hypertriglyceridemia with a high risk of pancreatitis that is unresponsive to standard lipid-lowering treatments.
- The use of metreleptin and volanesorsen might be considered subsequent to optimisation with standard antidiabetic and lipid-lowering therapies, if available, in the context of cost and risk–benefit evaluation, as the current evidence supporting their efficacy is based on small clinical trials with limited sample sizes and follow-up periods.
- Genetic counseling plays a crucial role and should be offered to all patients with confirmed or suspected genetic forms of FPLD. It enables informed decision-making regarding family planning, risk communication, and testing strategies. Following a confirmed diagnosis, cascade genetic screening of first-degree relatives is strongly recommended to identify asymptomatic carriers or undiagnosed cases and to enable early monitoring and intervention.
- It is imperative to consider the specific monitoring nuances for each subtype. Patients with FPLD2 require intensive cardiovascular follow-up owing to an elevated risk of cardiomyopathy and arrhythmias. Patients with FPLD3 require close hepatic monitoring owing to a higher prevalence of MASLD and more severe metabolic abnormalities. For FPLD1 and FPLD X, individualized follow-up is essential, contingent upon the severity of metabolic derangements.
- Women with FPLD, especially those with FPLD2 and FPLD3, require high-risk prenatal care, given the increased risk of obstetric complications, and contraceptive strategies should avoid oral estradiol formulations.
- Psychological support should be integrated into routine care to address the emotional and social impact of FPLD, particularly body image disturbances and stigma.
- Early diagnosis, comprehensive multidisciplinary management, personalized therapeutic strategies, and proactive genetic counseling are critical for improving the outcomes and quality of life in patients with FPLD.

## Limitations and future perspectives

Our proposed diagnostic criteria for FPLD were primarily based on expert consensus and observational data, which may limit their external validity. Nevertheless, the lack of standardized diagnostic tools and dependence on clinical expertise can lead to underdiagnosis or misclassification of FPLD, highlighting the importance of this consensus in supporting clinical decision-making.

An additional consideration is that certain therapeutic recommendations, particularly those pertaining to metreleptin and volanesorsen, are based on small randomized trials or uncontrolled studies. This underscores the need for more robust and generalizable evidence.

Expanding the BRAZLIPO registry presents a significant opportunity to enhance the characterization of the natural history of FPLD, validate the diagnostic criteria, and refine long-term follow-up strategies. Further research and methodological advancements are essential to thoroughly define genotype–phenotype correlations and to evaluate the comprehensive impact of preventive strategies. This would facilitate more meaningful cost-effectiveness analyses, and support the formulation of evidence-based policies and clinical recommendations.

Future research endeavors should prioritize the integration of centers and controlled clinical trials within the BRAZLIPO registry. This approach aims to assess the safety and efficacy of novel therapies, refine diagnostic methodologies, and improve early identification of affected individuals.

## Conclusions

FPLD represents a complex, severe, and heterogeneous condition characterised by variable loss of fatty tissue, leading to significant metabolic complications, including insulin resistance and cardiovascular diseases. The estimated prevalence of lipodystrophies varies widely, highlighting the urgent need for more rigorous diagnostic criteria to enhance identification and management of these patients. This expert consensus serves as a feasible guide based on the signs of lipoatrophy and metabolic abnormalities observed in the Brazilian population to improve clinical suspicion and facilitate early diagnosis of FPLD.

Through comprehensive screening of the multisystem manifestations and complications related to FPLD, we propose a therapeutic approach that encompasses lifestyle modifications, early interventions for comorbidities, and targeted pharmacological treatments. Additionally, therapies such as volanesorsen and metreleptin have shown promising efficacy in managing metabolic complications associated with lipodystrophies. Therefore, raising awareness of the clinical characteristics and optimising management with diagnostic and therapeutic strategies described in this consensus are crucial for improving the quality of life and prognosis of patients with this condition.

## Data Availability

No datasets were generated or analysed during the current study.

## Abbreviations

BMI: Body mass index
CAD: Coronary atherosclerotic disease
CPK: Creatine phosphokinase
DPP-4: Dipeptidyl peptidase-4
DXA: Dual-energy X-ray absorptiometry
FPLD: Familial partial lipodystrophies
FPLD1: FPLD type 1
FPLD2: FPLD type 2
FMR: Fat mass ratio
HDL: High-density lipoprotein
HIV: Human immunodeficiency virus
MASLD: Metabolic-associated steatosis liver disease
MASH: Metabolic dysfunction-associated steatohepatitis
NGS: Next-generation sequencing
OMIM: Online Mendelian Inheritance in Man
PL: Partial lipodystrophies
PPARG: Peroxisome proliferator-activated receptor gamma
PCOS: Polycystic ovary syndrome
SGLT2: Sodium-glucose cotransporter type 2
THE: Transient hepatic elastography
T2D: Type 2 diabetes
VLCD: Very low-calorie diets
WHR: Waist-to-hip ratio
